# Antibiogram Profiling of Antibiotics in Ear, Nose, and Throat Infections in Tertiary Healthcare Settings

**DOI:** 10.7759/cureus.54587

**Published:** 2024-02-20

**Authors:** Purnima Mariah Benedict Raj, Christy Joyliza Travasso, Raman Muthusamy

**Affiliations:** 1 Medical Microbiology, Saveetha Medical College and Hospital, Saveetha Institute of Medical and Technical Sciences (SIMATS) Saveetha University, Chennai, IND; 2 Center for Global Health Research, Saveetha Dental College and Hospital, Saveetha Institute of Medical and Technical Sciences (SIMATS) Saveetha University, Chennai, IND; 3 Center for Global Health Research, Saveetha Medical College and Hospital, Saveetha Institute of Medical and Technical Sciences (SIMATS) Saveetha University, Chennai, IND

**Keywords:** ent - ear nose and throat, antibiotic resistance, microorganism, healthcare, antibiotic sensitive testing

## Abstract

Introduction

Antibiotic resistance is an emerging threat in tertiary healthcare settings, with increased usage of antibiotics on patients having ear, nose, and throat (ENT) infections, the bacterial strains are becoming resistant to its treatment causing antibiotic resistance and ineffective treatment. This study focuses on the antibiogram profiling of bacterial pathogens by the conventional disc diffusion method in a tertiary healthcare setting and the recent method using a matrix-assisted laser desorption ionization time-of-flight mass spectrometry (MALDI-TOF) to identify bacterial strains isolated from infections of the ENT.

Materials and methods

Swab samples were collected from patients with ENT infections and were subjected to bacteriological and proteomic studies to assess the status of drug-resistant pathogens. About 125 samples were subjected to an antimicrobial susceptibility test by disc diffusion, and the bacterial isolates were screened on MALDI-TOF for identification.

Result

The study identified beta-hemolytic *Streptococci *as the most prevalent bacterial species, followed by *Pseudomonas aeruginosa* and *Staphylococcus aureus*. MALDI-TOF analysis yielded high identification accuracy for *beta-hemolytic Streptococcus pyogenes,* and the antibiogram profile of bacterial isolates indicated that most of the bacteria are resistant to penicillin, amoxicillin, and chloramphenicol.

Conclusion

The study emphasized the importance of appropriate antibiotic selection in treating ENT infections, considering local antibiograms and understanding antibiotic resistance patterns. This shall aid clinicians in choosing effective antibiotics, reducing treatment failure, and preventing the emergence of antibiotic resistance. Overall, the research provides valuable insights into antibiotic resistance in ENT infections.

## Introduction

Monitoring and surveillance of antibiotic resistance can help identify resistant strains and guide practitioners in selecting appropriate antibiotics. Antibiogram profiling is a laboratory technique that analyzes the sensitivity of bacterial isolates to various antibiotics. It is used to determine the sensitivity pattern of bacteria to different antibiotics and helps in choosing appropriate antibiotics for treatment. In the context of ENT infections, the microorganisms that inhabit the ear, nose, and throat (ENT) are diverse and closely related to one another. Some of these microbes are harmless in a typical normal condition [1].

The ENT are frequently the origin of infections due to frequent exposure to airborne microbes and direct environmental contact. ENT infections can have a variety of etiologies, including bacterial, viral, and fungal infections [2]. The etiology of the disease may sometimes be misinterpreted by the signs and symptoms. At times, the doctors find it increasingly challenging to identify the microorganisms causing the illness. Therefore, the doctor may promote the use of antibiotics regardless of the cause of the illness or follow the standard treatment guidelines without considering the antimicrobial susceptibility test (AST). However, this can lead to inappropriate use of antibiotics, which can contribute to antibiotic resistance [3]. Therefore, it is necessary to do antibiotic susceptibility tests to guide treatment and improve antibiotic selection [4].

Several studies show that *Staphylococcus aureus, Pseudomonas aeruginosa, Escherichia coli, Streptococcus pneumoniae* in otitis media and *S. pneumoniae* are the most commonly found bacterial species in cases of ENT infections with increasing resistance. The antibiogram helps guide empiric antibiotic therapy. With the prevalence of high levels of antimicrobial resistance to most of the commonly used antibiotics in ENT infections and recent reports on ampicillin and multidrug resistant (MDR) pathogens among the bacterial invaders, the need for prior antimicrobial testing and profiling of the antibiogram has become a necessity to prevent further complications [5-7]. Therefore, an antibiogram profiling is important to determine resistance in these infections for appropriate antibiotic selection in treatment in a tertiary care setting [8]. The objective of this research was to identify the most common bacteria that are resistant to different antibiotics in patients with ENT infections in hospitals and the antibiogram profile of isolated organisms. This serves as a guideline for the selection of appropriate antibiotics and medications for the treatment of these infections, which can greatly decrease the risk of treatment failure and the development of antibiotic resistance.

## Materials and methods

Sample collection

This cross-sectional hospital-based study was conducted at Saveetha Medical College and Hospital, Chennai, Tamil Nadu. All the patients diagnosed with ear, nose, and throat infections from the Department of Otolaryngology (ENT) were taken into consideration for this study. A total of 125 swab samples from the nose, throat, and ear were taken aseptically and transported to the microbiology lab within an hour of collection. Following all the stipulated ethical guidelines, ethical clearance was obtained from the college ethics committees (IRB No. 112101146).

Culture and identification

According to standard laboratory procedure, swabs were streaked into MacConkey (MAC) Agar and Mannitol Salt Agar (MSA) (HiMedia, India) and were incubated for 24 hours at 37°C [9]. After incubation, the bacterial colonies were isolated and transferred into an Eppendorf tube containing a viral transport medium. The sample tubes were stored in an ice-gel container to be transported to the Gujarat Biotechnology and Scientific Research Centre, Gandhinagar, Gujarat, for matrix-assisted laser desorption/ionization-time of flight (MALDI-TOF) screening. The bacterial isolates were inoculated onto a MALDI-TOF target plate, wherein the matrix solution α-cyano-4-hydroxycinnamic acid was applied and then allowed to air-dry. Target plates were examined using a MALDI-TOF mass spectrometer to identify the bacteria. The bacterial pathogens are identified using the concept of mass-to-charge ratios (m/z) present in bacterial proteins by MALDI-TOF screening.

Antimicrobial susceptibility 

An antimicrobial susceptibility test was performed by the Kirby-Bauer disc diffusion method to determine the resistance (R%) [10, 11]. The following drug discs were tested: penicillin (10 µg), amoxicillin (10 µg), cefixime (10 µg), cefpodoxime (10 µg), cefotaxime (10 µg), ceftriaxone (10 µg), erythromycin (10 µg), spiramycin (10 µg), clindamycin (10 µg), chloramphenicol (10 µg), levofloxacin (10 µg), and tetracyclines (10 µg). The above antibiotics were chosen in concordance with the prescription pattern followed at the study site. Then, a loopful of bacterial isolates from the above-cultured colony was taken and transferred into a tube containing nutrient broth, which was then swabbed onto Mueller-Hinton agar (MHA) plates using a sterile cotton swab. The antibiotic disc was placed on the agar plate and was incubated for 24 hours at 37°C. The antimicrobial activity was then determined by measuring zones of inhibition, and the collected data was analyzed using MS Excel (Microsoft Corporation, Redmond, Washington, United States). The significant differences between the samples were determined by p-values below 0.05 (p < 0.05) which were considered to be statistically significant [12].

## Results

MALDI-TOF screening

The screening of the bacterial isolate on MALDI-TOF indicated that the highest percentage of bacteria in the samples belonged mainly to the beta-hemolytic* Streptococci* species, specifically *Streptococcus pyogenes*, and the other commonly identified bacteria including* Streptococcus pneumoniae, Pseudomonas aeruginosa,* and *Staphylococcus aureus.*

Antibiotic-resistant profiles of bacterial isolates

A total of 125 swabs of ear, nose, and throat samples were collected from various nearby villages of Thiruvallur District. These samples were identified with the respective bacterial isolates: *Staphylococcus aureus*, *Pseudomonas aeruginosa*, beta-hemolytic S*treptococci*, and *Streptococcus pneumoniae *​​​​(Table 1).

**Table 1:** Samples collected from the ear, nose, and throat and identified bacterial isolates N: Number of samples collected

S. No.	Site of swabs collection (N = 125)	Staphylococcus aureus (N = 45)	Pseudomonas aeruginosa (N = 35)	Beta-hemolytic Streptococci (N = 25)	Streptococcus pneumoniae (N = 20)
	Ear (N = 30)	9	8	6	7
	Nose (N = 45)	15	13	8	9
	Throat (N = 50)	21	14	11	4

The results of disc diffusion testing revealed the susceptibility rates of isolated bacteria in the given Table 2. These bacteria had marked results for resistance and susceptibility against various antibiotics as listed. Out of the sample collected, all the bacterial isolates were most resistant to penicillin (N = 125) (15.2%), amoxicillin (N = 125) (15.2%), cefixime (N = 125) (7.2%), cefpodoxime (N = 125) (4.8%), cefotaxime (N = 125) (8.8%), ceftriaxone (N = 125) (4%), erythromycin (N = 125) (15.2%), spiramycin (N = 125) (5.6%), clindamycin (N = 125) (10.4%), and chloramphenicol (N = 125) (11.2%). Higher resistance was seen in *S. aureus*, which is resistant to clindamycin (N = 11) (24.44%), cefotaxime (N = 8) (17.77%), and amoxicillin (N = 7) (15.5%); *P. aeruginosa* isolates were resistant to erythromycin (N = 8) (22.85%), clindamycin (N = 7) (20) and cefixime (N = 6) (17.4%). Beta-hemolytic *Streptococci* revealed the highest resistance to chloramphenicol (N = 5) (20%), erythromycin, and penicillin (N = 3) (12%). *Streptococcus pneumoniae* is most resistant to amoxicillin, erythromycin (N = 5) (25%), and cefpodoxime (N = 2) (10%).

**Table 2:** Antibiotic resistance profile of an isolated bacteria from ENT infection R%: percent of isolates resistant to an antibiotic drug

Antibiotic tested	Staphylococcus aureus (N = 45) R%	Pseudomonas aeruginosa (N = 35) R%	Beta-hemolytic streptococci (N = 25)R%	Streptococcus pneumoniae (N = 20) R%
Penicillin	5 (11.11)	4 (11.4)	3 (12)	2 (10)
Amoxicillin	7 (15.5)	2 (5.71)	5 (20)	5 (25)
Cefixime	0	6 (17.14)	2 (8)	1 (5)
Cefpodoxime	0	3 (8.57)	0	2 (10)
Cefotaxime	8 (17.77)	0	1 (4)	2 (10)
Ceftriaxone	4 (8.88)	0	0	0
Erythromycin	3 (6.66)	8 (22.85)	3 (12)	5 (25)
Spiramycin	2 (4.44)	0	2 (8)	1 (5)
Clindamycin	11 (24.44)	7 (20)	1 (4)	0
Chloramphenicol	5 (11.11)	5 (14.2)	5 (20)	2 (10)

## Discussion

Antibiotics have become less effective against infectious strains, leading to increased hospitalizations and treatment failures. Resistant pathogens like *Staphylococcus aureus, Mycobacterium tuberculosis,* and *Escherichia coli* are causing concern in tertiary healthcare settings [7]. In this study, the common bacteria found in the bacteriological sample collected from ENT infection patients were beta-hemolytic *Streptococcus, Streptococcus pneumoniae, Pseudomonas aeruginosa*, and *Staphylococcus aureus*. This study aligns with other ENT studies that consistently identified beta-hemolytic *Streptococcus* as resistant to antibiotics. With the emergence of multidrug resistance even to the standard antimicrobials, including bacteria and fungal pathogens, the use of empirical therapy has led to high resistance among the bacterial and fungal pathogens inhabiting the ENT. As reported in a recent study, *Streptococcus pyogenes *was the predominant bacterial pathogen (30%) of pharyngitis in pediatric patients in tertiary care hospitals in Germany [12, 13]. However, the distribution of species may vary depending on factors like location and patient demographics [14].

Moreover, the identification accuracy of *Streptococcus pyogenes *using MALDI-TOF is consistent with the specificity and reliability of other standard methods undertaken in earlier studies [15]. Hospitals use a wide range of antibiotics, starting with third-generation cephalosporins like cefixime, cefpodoxime, cefotaxime, and ceftriaxone [16]. These antibiotics are known for their activity against pathogens causing infections. However, prolonged use of these antibiotics has been linked to the development of resistance. In this study, we aimed to investigate the antibiogram profiles of antibiotics used in ENT infections in a tertiary healthcare setting from patients with ENT infections, which revealed that most of the bacterial isolates were resistant to penicillin, amoxicillin, cefixime, cefpodoxime, cefotaxime, erythromycin, spiramycin, clindamycin, and chloramphenicol, with the highest resistance toward penicillin and chloramphenicol. However, the findings of this study align with the previous study conducted on throat infection and with the report on the emergence of methicillin, third-generation cephalosporin, and chloramphenicol. Due to the frequent therapeutic options in tertiary hospitals, public health concerns have increased in LMIC countries as per the study conducted in Nigeria [6,17].

Similarly, in a study carried out by Yitayeh et al. in Ethiopia between 2015 and 2018, 18.7% of clinical specimens were tested for aerobic bacterial pathogens by the disc diffusion method, with a higher positivity in ear swab samples, as observed in the present study [18]. The prevalence of infection was highest in females. *Escherichia coli *and *Klebsiella* species were the predominant bacterial isolates, with 61.8% of the isolates being MDR. Gram-positive cocci demonstrated resistance against norfloxacin, ciprofloxacin, and clindamycin, while Gram-negative bacteria showed resistance against ampicillin and penicillin. The research study denotes a high number of MDR isolates in low-income countries, emphasizing the need for clinicians to make rational antibiotic choices and use antimicrobial susceptibility testing for effective treatment.

These findings can assist healthcare professionals in selecting appropriate antibiotics for treating infections caused by the bacterial strains observed in the study [19]. Such targeted antibiotic therapy can improve treatment outcomes, minimize the risk of treatment failure, and combat the development of antibiotic resistance. Overall, the utilization of both MALDI-TOF and disc diffusion methods in this study contributes to a comprehensive understanding of the bacterial species and their resistance profiles in the samples tested. These findings are critical in formulating effective treatment regimens while implementing measures to control antibiotic resistance. This will lead to improved patient care and management of ENT infections.

## Conclusions

The bacteria with the highest incidence were beta-hemolytic *Streptococcus* and *Streptococcus pneumoniae*, followed by *Pseudomonas aeruginosa* and *Staphylococcus aureus*. The antibiogram profile of clinical samples revealed that these bacteria showed the highest inhibition against beta-lactam antibiotics such as penicillin and cephalosporins. The highest rate of resistance by the causative agents was against chloramphenicol, penicillin, and amoxicillin antibiotics. Hence, the use of the antibiotics ceftriaxone, cefpodoxime, and cefotaxime, which inhibit the growth of some of the bacterial isolates compared to others, is suggested to be implemented in treatment. The findings contribute to the understanding of bacterial species and resistance patterns, aiding clinicians in choosing effective antibiotics, reducing treatment failure, and preventing the emergence of antibiotic resistance.

Limitations

The study is based on ear, nose, and throat swab samples collected from Saveetha Medical College and Hospitals, Chennai, so the sample size was the major limitation. The antibiogram profile of microbes and their possible antibiotic treatment is specific to Tiruvallur District, Chennai, and not the whole city of Chennai. 
